# Laparoscopic Sleeve Gastrectomy with Omentopexy: Is It Really a Promising Method?—A Systematic Review with Meta-analysis

**DOI:** 10.1007/s11695-021-05327-8

**Published:** 2021-03-06

**Authors:** Piotr Zarzycki, Jan Kulawik, Piotr Małczak, Mateusz Rubinkiewicz, Mateusz Wierdak, Piotr Major

**Affiliations:** 2nd Department of General Surgery, Jagiellonian University Medical College, Jakubowskiego 2 st., 30-688 Krakow, Poland

**Keywords:** Systematic review, Meta-analysis, Bariatric surgery, Laparoscopic sleeve gastrectomy, Omentopexy

## Abstract

**Purpose:**

Laparoscopic sleeve gastrectomy (LSG) is one of the most commonly performed bariatric procedure worldwide. Omentopexy during LSG is a novel variation of this well-established technique. There are no clear conclusions on indications for this procedure, safeness, and effects of such a method. We aimed to compare the outcomes of laparoscopic sleeve gastrectomy (LSG) with omentopexy (OP) and without omentopexy.

**Materials and Methods:**

We searched the Medline, EMBASE, and Scopus databases up-to June 2020. Full-text articles and conference abstracts were included for further analysis. This review follows the PRISMA guidelines.

**Results:**

Of initial 66 records, only 4 studies (*N* = 1396 patients) were included in the meta-analysis. Our findings showed that LSG with omentopexy had significantly lowered overall morbidity compared to LSG without omentopexy (RR = 0.38; 95% CI [0.15, 0.94]; *p*=0.04). Gastric leakage rate (RR = 0.17; 95% CI [0.04, 0.76]; *p* = 0.02) was also significantly lower in LSG with omentopexy. There were no significant differences between groups in length of hospital stay.

**Conclusions:**

Our meta-analysis showed that LSG with omentopexy may be a feasible procedure for decreasing morbidity and gastric leak rate. However, despite promising results, the procedure needs to be researched more in randomized controlled studies to draw solid conclusions.

**Supplementary Information:**

The online version contains supplementary material available at 10.1007/s11695-021-05327-8.

## Introduction

The problem of severe obesity affects a large part of society. According to WHO report, in 2016, 39% of the adult population was overweight and 13% suffered from severe obesity [1, 2]. Bariatric procedures are the most effective treatment [3]. The effect of weight loss is long-lasting and its positive impact on the treatment of comorbidities (hypertension, type 2 diabetes) is also observed [4]. Laparoscopic sleeve gastrectomy is one of the most commonly performed bariatric procedures worldwide [5]. Apart from the obvious positive metabolic and restrictive aspects, LSG procedure is not without its potential complications. Bleeding from the staple line, gastric leakage, and gastroesophageal reflux are the most frequent and common complications after LSG [6, 7]. This method is constantly evolving and despite its simplicity, we are still collecting data on many different technical aspects of the procedure. There are ongoing discussions about the diameter of the calibration probe, staple line reinforcement, or the distance of the resection line from the pylorus. All these considerations are intended to achieve better treatment outcomes in terms of both greater weight reduction and decreased risk of complications. Omentopexy is a novel technique performed during laparoscopic sleeve gastrectomy. In general, in the method, the remnant stomach is fixed to the gastrosplenic and gastrocolic ligaments [8]. This procedure was added to classic laparoscopic sleeve gastrectomy to decrease the rate of gastroesophageal reflux, postoperative food intolerance, and gastric leaks. Arslan et al. suggest that omentopexy stabilizes the posterior stomach wall and can prevent the gastric twist, which is a functional cause of gastric stenosis [9]. Gastric volvulus (meaning when part of the stomach after sleeve resection rotates around the anatomic axes) may be partially responsible for the occurrence of complications. Laparoscopic sleeve gastrectomy (LSG) with omentopexy (OP) has been performed for a few years; however, there is still no consensus on the indication for it. Available literature on the safety and effects of this novel technique is still sparse. Our study aimed to evaluate different aspects of laparoscopic sleeve gastrectomy with omentopexy in regard to morbidity, gastric leaks, and length of hospital stay (LOS). To our best knowledge, there is no meta-analysis in the literature comparing LSG with or without omentopexy (OP).

## Methods

### Search Strategy

A search was conducted by two researchers (JK and MR) in June 2020 of Medline, Embase, and Scopus, with no language restriction and using the search terms: “omentopexia,” “omentopexy,” “omentum reinforcement,” “omentum fixation,” “omentum suturing,” and combinations of these with: “LSG,” “sleeve gastrectomy” using the Boolean operators “AND” and “OR.” Detailed search strategy for OVID is available in supplementary file 1.

Inclusion criteria were (1) comparison of sleeve gastrectomy with and without omentopexia and (2) reporting of gastric leak rate. The papers included had to be either a randomized controlled trial (RCT) or a comparative study with a control group. Conference abstracts were also considered when contained an appropriate amount of data on complication rate or gastric leak rate for each group. All criteria mentioned above were required to enroll a study for further evaluation. The exclusion criteria were as follows: (1) lack of comparative data; (2) lack of primary outcomes or insufficient data to analyze.

### Data Extraction and Quality Assessment

All references were reviewed and evaluated independently by two researchers (PZ and MW). In case of any doubts about eligibility for inclusion, an attempt was made to reach consensus. If no resolution was possible, an arbitrary decision was made by another reviewer. Data from included studies were extracted independently. When available, the following data were extracted: first author, year of publication, country, number of operated subjects, and outcomes of interest.

Randomized study quality and risk of bias were assessed using The Cochrane Collaboration’s tool for assessing risk of bias. Non-randomized studies were evaluated according to the Newcastle–Ottawa Scale (NOS), which consists of three factors: patient selections, comparability of study groups, and assessment of outcomes. A score of 0 to 9 was assigned to each study, and studies achieving a score of 6 or higher were considered high quality [10]. This study was performed according to the Preferred Reporting Items for Systematic Reviews and Meta-Analyses (PRISMA) guidelines and Meta-Analysis of Observational Studies in Epidemiology (MOOSE) consensus statement [11, 12].

### Outcome Measures

The primary outcome measure of this systematic review was gastric leakage. Secondary outcome measures were overall morbidity and length of hospital stay (LOS).

### Statistical Analysis

The analysis was performed using RevMan 5.3 (freeware from the Cochrane Collaboration). Statistical heterogeneity and inconsistency were measured using Cochran’s Q tests and *I*^2^, respectively. Qualitative outcomes from individual studies were analyzed to assess individual and pooled risk ratios (RR) with pertinent 95% confidence intervals (CI) comparing omentopexia LSG with standard LSG, and by means of the Peto fixed effects method in the presence of low or moderate statistical inconsistency (*I*^2^ ≤ 10%), and by means of a random-effects method (which better accommodates clinical and statistical variations) in the presence of high statistical inconsistency (*I*^2^ > 10%). Weighted mean differences (WMD) with 95% CI are presented for quantitative variables using the inverse variance fixed effects or random effects method. Statistical significance was observed with two-tailed 0.05 level for hypothesis and with 0.10 for heterogeneity testing, while unadjusted *p*-values were reported accordingly. This study was performed according to the Preferred Reporting Items for Systematic reviews (PRISMA) guidelines and MOOSE consensus statement.

## Results

**Fig. 1:**
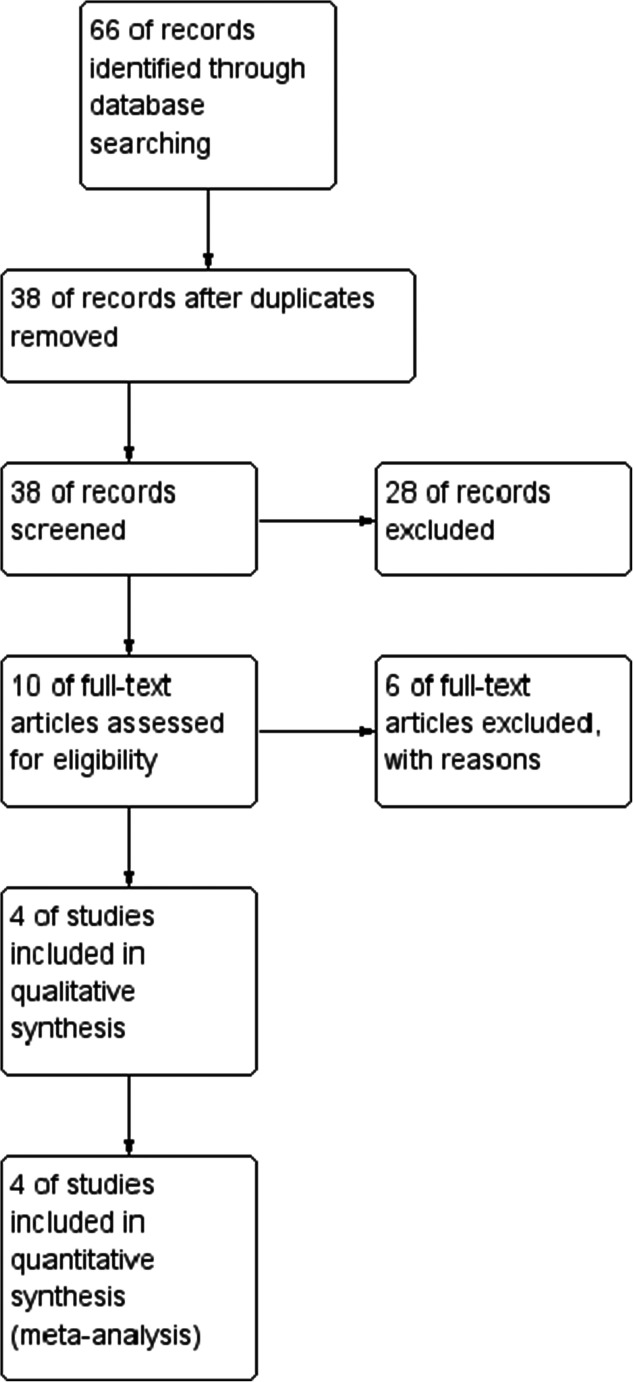
PRISMA flowchart

The initial search yielded 66 records. After removal of the duplicates and abstract screening, we selected 4 studies. To provide as vast data as possible, we decided to include conference abstracts as well (Table 1). The study group covered 703 patients with omentopexy and 693 without omentopexy (1396 patients in total). The PRISMA flowchart is presented in Fig. 1.

**Table 1:** Baseline characteristics of included studies

Study	Year	Design	*N* with/without omentopexy	Morbidity	LOS (days)	Gastric leaks	Full text	NOS
Pilone [13]	2019	RCT	96/90	3/18	4.5/5.8	0/3	Yes	
Sharma [14]	2020	CC	370/367	6/16	ND	0/7	Yes	6/9
Abdo [15]	2014	CC	87/86	6/7	ND	0/1	No	N/A
Hassan [16]	2018	C	150/150	ND	1.33/1.67	0/1	no	N/A

*C*, cohort; *CC*, case-control; *RCT*, randomized control study; *ND*, no data; *LOS*, length of stay; *NOS*, Newcastle–Ottawa Scale; *N*, number

**Fig. 2:**
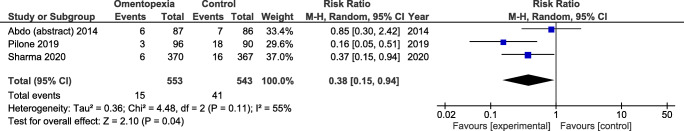
Overall morbidity analysis

Morbidity was reported by three authors, including *N* = 1096. There were significant differences in favor of LSG with omentopexy; RR = 0.38; 95% CI [0.15, 0.94]; *p*=0.04 (Fig. 2). However, the sample size was small. The heterogeneity between studies was moderate, *I*^2^=55%.

**Fig. 3:**
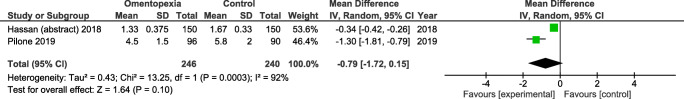
Length of stay analysis

Length of stay was reported by two authors, including *N* = 486 patients. There was no significant difference between group: MD = –0.79; 95% CI [−1.72, 0.15]; *p* = 0.10 (Fig. 3). However, heterogeneity between studies was significant, *I*^2^ = 92%.

**Fig. 4:**
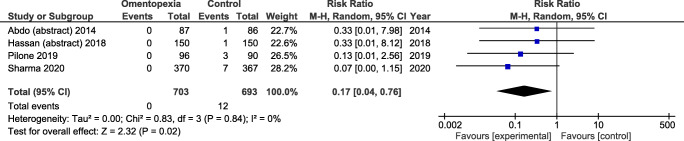
Gastric leak rate analysis

Gastric leakage was reported by four authors, including *N* = 1396 patients. There were significant differences in favor of LSG with omentopexy: RR = 0.17; 95% CI [0.04, 0.76]; *p* = 0.02 (Fig. 4). Heterogeneity between studies was low, *I*^2^ = 0%.

There were no differences in BMI in particular studies between LSG with and without omentopexy.

## Discussion

Our systematic review and subsequent meta-analysis, including a total of 1396 patients (703 LSG with OP), showed that laparoscopic sleeve gastrectomy with omentopexy is associated with significantly lower morbidity and lower gastric leak rate. Batman et al., in retrospective study with 1200 patients who underwent sleeve gastrectomy and omentopexy, suggest that it is a safe procedure with low complication rates (1.33% of all patients) [17]. Only one of the included studies in our meta-analysis was a randomized control study (Pilone et al.).

The most severe complication after LSG is gastric leakage, which is associated with higher mortality rate [18]. Our study shows that LSG with omentopexy is related with significantly lower rate of gastric leakage. Leaks occur when the intraluminal pressure is higher than the strength of the staple line [19]. Sharma et al. give mathematical and anatomical theoretical explanations about increasing intragastric pressure after LSG. After standard LSG, the medial forces acting on sleeved stomach from ligaments are stable, but the lateral forces are lost as a result of detachment of the greater omentum. Omentopexy recreate stomach stabilization inside the abdomen preventing it from kinking and thus reducing the intragastric pressure [14].

Few studies, carried out on a large group of patients, despite being excluded from our meta-analysis, confirm that omentopexy reduces the risk of gastric leakage. Sabri et al., in a retrospective cohort study on 2000 patients, shown that LSG with omentopexy can be effective in decreasing staple line bleeding, leakage, and hospital stay, although it prolongs the operative time [20]. Lale et al., in study of 3942 LSGs divided into 3 groups ( group 1: no reinforcement, group 2: staple line reinforcement with fibrin glue, group 3: staple line reinforcement with omentopexy (SLR-O)), show that SLR-O during laparoscopic sleeve gastrectomy is a promising method for the prevention of postoperative leakage, bleeding, and twist complications with an increase in the duration of operation [21]. However, the authors point out that taking into account the advantages of the introduced method, the extension of the duration of the procedure is acceptable.

In addition, only two selected studies (Pilone et al. and Hassan et al.) report length of procedure which longer in groups with omentopexy in both studies. There was no statistical analysis on the influence of omentopexy on the duration of procedure. However, despite the longer time with omentopexy, it may be worth considering if it will be proven that it reduces the number of leaks after standard LSG.

Analyzed studies did not provide any additional information on whether patients required any subsequent revision surgery. In our opinion, omentopexy may impede the procedures of revision bariatric surgery in the future*.*

Complication rate after laparoscopic sleeve gastrectomy is associated with higher body weight and Body Mass Index (BMI) [22]. Referring to the results of our study, it is worth adding that despite the lower number of complications after LSG with omentopexy, one study showed different results. Ricardo et al., in a single-center study, in a group of 181 patients who underwent a laparoscopic sleeve gastrectomy (41% also underwent omentopexy), showed that omentopexy performed in patients who are super-obese had more complications than severely obese patients. Based on the data presented by Major et al. and Ricardo et al., qualification for LSG with omentopexy in super-obese patients should be more thoughtful due to a significantly higher number of complications [22, 23].

The length of stay is a suitable indicator of the patient’s full mobilization, appropriate oral fluid tolerance, and whether there have been complications that may have contributed to delayed discharge. According to the ERAS Society guidelines, the patient after bariatric surgery may be discharged home when he meets the following criteria: tolerating an oral diet, consuming at least 1000 ml of fluids a day, no need for intravenous fluid therapy, postoperative pain is controlled with oral medications, the level of physical activity is similar to that before surgery, has constant contact with the treatment center, and there were no complications that would require postponement of hospitalization. Our study also shows that there is no significant difference in LOS between LSG with omentopexy and LSG without omentopexy. Heterogeneity between studies was significantly high (l2 92%). We observed that there is a noticeable difference in time range: Pilone et al. reported LOS in days (4.5 versus 5.8), but Hassan et al. reported in hours (32+/−9 versus 40+/−8) [13, 16]. The difference may depend on the method of calculating the length of stay in the hospital and on well-developed outpatient care in accordance with the ERAS protocol [24, 25]. In addition, an unbiased comparison of LOS between studies is difficult because it can be associated with local customs rather than fulfilling clear objective discharge criteria. Nevertheless, there is no information about the criteria for home discharge in both studies.

Our meta-analysis shows that omentopexy may decrease gastric leaks after LSG. However, omentopexy is not the first technique used to minimize this complication. Various preventive gastric leak techniques have been proposed, such as staple-line reinforcement (SLR). Another technique used for preventing staple-line leaks involves oversewing sutures. No consensus has been reached in the literature on the efficacy of SLR after sleeve gastrectomy in preventing leaks [26]. For example Demeusy et al. in his analysis of total 198339 primary LSG and the relationship between various SLR techniques demonstrated that SLR is associated with decreased rates of bleeding and reoperations but does not affect leak rates [27]. On the other hand, Gagner et al. in his systematic review of staple-line leaks following LSG demonstrated a significantly lower rate using APM (absorbable polymer membrane) staple-line reinforcement as compared to oversewing, use of sealants, BPS reinforcement, or no reinforcement [28].

Two studies (Pilone et al. and Sharma et al.) mentioned about the effectiveness of SLR or fibrin glue suture on decreasing postoperative bleeding. Pilone et al. hypothesize that NBCA+MS sealant (Glubran®2) may decrease the risk of staple line bleeding and fixing of the omentum can enhance adhesive properties, reducing risk of leak. In Sharma’s study, the rate of bleeding did not reach statistical significance, in contrast to significantly lower leakage rates with omentopexy as compared with no omentopexy. According to the literature, there is also no consensus about the influence of fibrin glue on decreasing bleeding. In 2014, Musella et al., in randomized control trial, showed that the use of fibrin sealant in LSG significantly reduces postoperative bleeding [29]. In turn, Mehmet Bayrak et al. suggest that the use of fibrin glue and over-sewing for staple line reinforcement during laparoscopic sleeve gastrectomy did not affect postoperative or perioperative hemorrhage and leakage [30].

In addition to the above-mentioned conclusions, some researchers compare the effect on reducing postoperative gastroesophageal reflux (GER) and food intolerance. On the one hand, Filho et al. show that LSG with omentopexy improved the clinical score of GER and did not cause significant changes in the lower esophageal sphincter (LES) tone [8]. On the other hand, Nasrati et al., in a retrospective cohort study in a group of 201 patients, show that omentopexy does not have a significant effect on reducing the incidence of de novo GERD after LSG after 1-year observation [31]. Also Cheguevara et al., in a prospective randomized controlled trial in a group of 60 LSGs divided into two groups, show that omentopexy did not significantly decrease postoperative food intolerance or GI symptoms in morbidly obese patients undergoing LSG. Authors suggest that GERD impact scores were low at all the measured time points with or without an omentopexy and it may be related to the fact that all patients had administered a proton-pump inhibitor for at least 3 months [32]. So far, no unequivocally positive effect on the reduction of symptoms in the form of gastroesophageal reflux has been found, but the described stabilization of sleeved gastric tube caused by omentopexy and its effect on facilitating food passage after resection requires further research and observation.

Our study has a lot of limitations. Two of four included studies are conference abstracts. In the included studies, the number of patients with gastric leakage was low. For this reason, to achieve sufficient patient numbers, larger multicenter studies are required.

Omentopexy is not a standardized procedure and the technique description varies between the studies*.* Sharma et al., in their technique, placed 2–4 sutures at the site proximal to incisura and one suture at the most distal end of staple line [14]. Pilone et al., after formation of the sleeve, applied a layer of the synthetic sealant on all rime sutures and cover it by an omentum flap [13]. In Batman et al., omentopexy is performed by suturing the omentum back to the greater curvature with V-Loc sutures along the entire staple line [17]. Furthermore, in two studies, there is a difference between the method of suture line reinforcement before performed omentopexy (Pilone et al. use a synthetic sealant and Sharma et al. use a buttress material (BSLR)). Despite that, in both cases, groups with omentopexy have lower numbers of gastric leakage*.* The above examples show the difficulty in comparing procedures performed with different techniques. The lack of a clearly defined surgical technique affects the diversity of the results obtained.

In two studies, Pilone et al. and Sharma et al. used similar size of the boogie for sleeve calibration (42–48 Fr vs. 40–44 Fr). The potentially larger difference in the diameter of the boogie may affect the number of leaks. In literature, the size of the boogie, used for calibration, is also a subject of controversies, ranging between 32 and 60 Fr. Aurora et al., in a large systematic review (4888 patients), suggested that larger boogie size may decrease the leak rate [33].

One study (Sharma et al.) includes information about dividing the team into surgeons doing LSG only, LSG with omentopexy only, or surgeons doing both procedures. In this particular case, all procedures were done by three surgeons (surgeon A performs LSG with omentopexy, while surgeons B and C do not perform omentopexy). There are no variables in the form of a different experience, skills, or a different position on the learning curve.

The effect of SLR after sleeve gastrectomy on bleeding or gastric leakage is still a controversial topic. Studies show either no effect or extremely different effects on bleeding and leak or no effect on leakage but decreased staple line bleeding. There is a need for more research to confirm the benefits of procedures potentially reducing gastric leakage or bleeding after LSG.

Because of limitations, included studies still do not provide decisive, high-grade results. In our opinion, more studies, especially RCTs, are required to fully assess this approach because the available data are of limited quality.

## Conclusions

The results of our study show that LSG with omentopexy can be a feasible procedure for decreasing morbidity and gastric leak rate. There is no significant difference between lengths of stay. Some studies show potential impact of reducing postoperative gastrointestinal reflux. It is an interesting procedure with promising results but there is still a lack of well-designed high-quality studies.

## Supplementary Information

ESM 1 (PNG 45 kb)
