# Supplementary figures and images for: Laparoscopic Sleeve Gastrectomy with Omentopexy: Is It Really a Promising Method?—A Systematic Review with Meta-analysis
Source: Obes Surg. 2021 Mar 6;31(6):2709–16. doi: 10.1007/s11695-021-05327-8 (PMC8113139; doi:10.1007/s11695-021-05327-8)

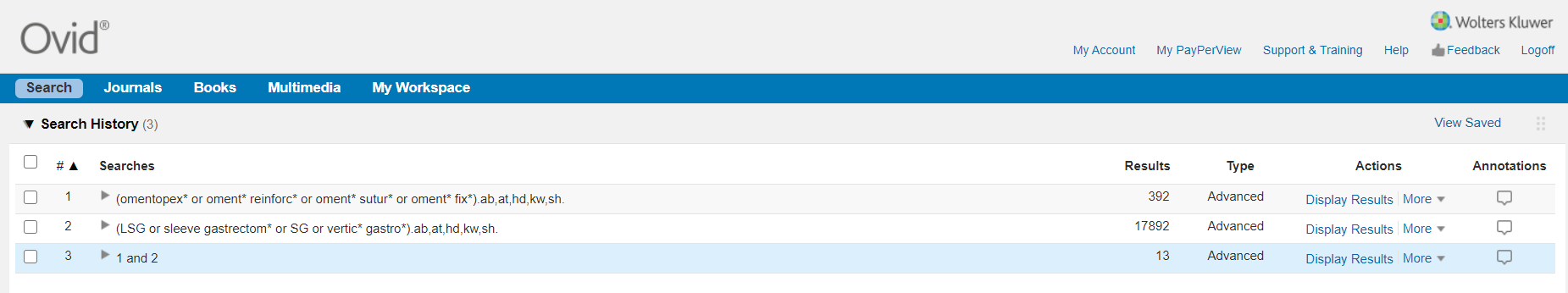

Supplement: Supplementary file 1 — (PNG 45 kb) [file 11695_2021_5327_MOESM1_ESM.png]
